# IMPROVING THE PERSONAL CARE ASSISTANT WORKFORCE THROUGH TRAINING, DATA, AND TECHNOLOGY

**DOI:** 10.1093/geroni/igz038.2467

**Published:** 2019-11-08

**Authors:** Fuad Abujarad, Sarah J Swierenga, Clare Luz, Katherine Hanson, Chris Curtin, Zhehui Luo, Lauren A Swanson-Aprill

**Affiliations:** 1 Yale University, New Haven, Connecticut, United States; 2 Michigan State University, East Lansing, Michigan, United States; 3 DIRECT CARE PROFESSIONAL TRAINING, Traverse City, Michigan, United States; 4 Department of Epidemiology and Biostatistics, Michigan State University, East Lansing, Michigan, United States; 5 Michigan Department of Health and Human Services, Aging and Adult Services Agency (AASA), Lansing, Michigan, United States

## Abstract

Ensuring a quality personal care aide (PCA) workforce is critical to meeting the needs of an aging population. The Integrated Model for Personal Assistant Research and Training (IMPART) program is a PCA training and advocacy model designed to increase the number of qualified PCAs. A core component is an evidence-based, comprehensive PCA training program, Building Training…Building QualityTM (BTBQTM), supported by a robust data platform. This online system was designed to register, train and certify PCAs and Trainers, maintain a qualified PCA workforce database, and enhance the capacity to track IMPART process, performance, and impact measures. This innovative system was developed using a User-Centered Design approach, which includes four main user interfaces: Administrator, PCA, Trainer, and Public. It automates central administration functions, tracking, and reporting of training events, and standardizes and consolidates all training activities on a scalable and usable web-based technology platform. User acceptance testing of the tool and a usability evaluation with representative PCAs and trainers has been completed. Over 50 PCAs have completed BTBQTM modules and 22 new trainers have completed a new BTBQTM Trainer Certificate program during the tool development phase. The PCAs are already reporting substantially higher wages. Launching the new web-based data platform in April 2019 will make it possible for these programs to scale up for wide distribution. As more PCAs and trainers graduate, the number of qualified PCAs will increase exponentially. The data collected with this technology can inform responsible fiscal and policy decisions about resource allocation to support a stronger PCA workforce.

